# 

**Published:** 1882-12

**Authors:** 


### TABLE OF CONTENTS FOR DECEMBER, 1882.

### ORIGINAL COMMUNICATIONS.

ART. I.—Operation for Strabismus Completed under Narcotism of the Bromide of Ethyl in Fifty-two Seconds for Ethylization and Tenotomy. By JULIAN J. CHISOLM, M. D. .... 625
ART. II.—Some Observations on the Use of the Tampon in the Hemorrhage of Abortion. By RUFUS W. GRISWOLD, M. D. .... 629

### SELECTIONS.

On the Microbe of Typhoid Fever .... 634
The Army Medical Museum and Library .... 637
The Latest about Bacteria .... 638
One of the Vagaries of the Anti-Vaccinationists .... 639
Diphtheria .... 640
Health of Criminal Women .... 641
A Novel use for Pepsin .... 642
Viburnum Prunifolium .... 642
Spermatorrhœa and Hypochondriasis .... 642
The "Raspberry" Vaccination Scar, and its Guarantee of Protection from Small Pox .... 642
Constipation in Females .... 644
Look out for Small Pox .... 644
Diagnosis of Fœtal Monstrosities .... 644
Heredity of Sebaceous Cysts .... 645
Swallowing Artificial Teeth .... 645
Reckless Midwifery .... 646
Undeveloped Testes from Tobacco-Chewing .... 646
A Heavy Brain .... 646
An Important Medico-Legal Decision .... 647
EDITORIAL .... 648
CURRENT NEWS AND OPINION .... 650
BIBLIOGRAPHICAL .... 652
POPULAR SCIENCE DEPARTMENT .... 653

